# Multi-behavioral obesogenic phenotypes among school-aged boys and girls along the birth weight continuum

**DOI:** 10.1371/journal.pone.0212290

**Published:** 2019-02-21

**Authors:** Andre Krumel Portella, Catherine Paquet, Adrianne Rahde Bischoff, Roberta Dalle Molle, Aida Faber, Spencer Moore, Narendra Arora, Robert Levitan, Patricia Pelufo Silveira, Laurette Dube

**Affiliations:** 1 Desautels Faculty of Management, McGill Center for the Convergence of Health and Economics, McGill University, Montreal, QC, Canada; 2 PostGraduate Program in Pediatrics, Universidade Federal de Ciencias da Saude de Porto Alegre, Porto Alegre, RS, Brasil; 3 School of Health Sciences, Centre for Population Health Research, University of South Australia, Adelaide, South Australia, Australia; 4 Division of Neonatology, Department of Pediatrics, University of Toronto, Toronto, ON, Canada; 5 Programa de Pós-Graduação em Saúde da Criança e do Adolescente, Faculdade de Medicina, Universidade Federal do Rio Grande do Sul, Porto Alegre, RS, Brazil; 6 Arnold School of Public Health, University of South Carolina, Columbia, SC, United States of America; 7 The INCLEN Trust, New Delhi, India; 8 Institute of Medical Science, University of Toronto, Toronto, ON, Canada; 9 Centre for Addiction and Mental Health (CAMH), Toronto, ON, Canada; 10 Department of Psychiatry, Faculty of Medicine, McGill University, Montreal, QC, Canada; 11 Sackler Institute for Epigenetics & Psychobiology, McGill University, Montreal, QC, Canada; 12 Ludmer Centre for Neuroinformatics and Mental Health, Douglas Hospital Research Centre, Montreal, QC, Canada; Purdue University, UNITED STATES

## Abstract

Evidence shows that extremes of birth weight (BW) carry a common increased risk for the development of adiposity and related cardiovascular diseases, but little is known about the role of obesogenic behaviors in this process. Moreover, no one has empirically examined whether the relationship between BW, obesogenic behaviors and BMI along the full low-to-high birthweight continuum reflects the U-shape pattern expected from common risk at both BW extremes. Our objective was to characterize physical activity, screen time, and eating behavior and their relationship to BMI as a function of BW among school-aged boys and girls. In this cross-sectional study, 460 children aged 6 to 12 years (50% boys) from Montreal, Canada provided information on sleeping time, screen time, physical activity levels, eating behavior (emotional, external and restrained eating) and anthropometrics (height, weight, BW) through parent reported questionnaires. BMI was normalized using WHO Standards (zBMI), and BW expressed as ratio using Canadian population standards (BW for gestational age and sex). Analyses were conducted using generalized linear models with linear and quadratic terms for BW, stratified by sex and adjusted for age, ethnicity and household income. In boys, physical activity and screen time showed U-shaped associations with BW, while physical activity had an inverted U-shaped in girls. Emotional and restrained eating had positive linear relations with BW in boys and girls. Sleep time and external eating were not associated with BW. A U-shaped relationship between BW and zBMI was found in boys but no association was found in girls. Only sleep (in boys and girls), and emotional eating (girls only) were related to zBMI and mediation of the BW-zBMI relationship was only supported for emotional eating. In conclusion, BW relates to obesogenic behaviors and BMI in both non-linear and linear ways, and these associations differed by sex.

## Introduction

Both extremes of the birth weight normal distribution have been associated with higher incidence of later life chronic diseases and conditions such as increased adiposity, metabolic syndrome, dyslipidemia, and cardiovascular disease[1–3]. Although the literature has extensively explored this relationship in terms of the programming of metabolic functions[4, 5], there is also emerging evidence for the early-life programming of behavioral phenotypes such as physical activity[6, 7], feeding preferences[8], and sleep patterns[9].

Low birth weight individuals, for instance, have been found to have food preferences biased towards highly caloric, highly palatable foods[10–12], be more impulsive towards a sweet reward[13], have decreased sensitivity to the enjoyment of the sweet taste[14, 15], and also exercise less[16, 17]. In contrast, much less evidence is available for the impact of high birth weight on behavior in human research. We have recently reported a negative correlation between birth weight and preference for fat in the habitual dietary intake of 6–12 years boys, and Ester et al[18]showed at high birth weight children scored higher on food approach and lower on food avoidant scales. Animal models suggest a similar pattern of behavioral alterations[16, 17, 19, 20]. In general, boys and girls differ both at the neurological and biological levels in terms of timing of fat rebound, puberty, and body composition [21], and these facts ultimately influence feeding behavior [22, 23]. Therefore, while prenatal programming seems to modify eating patterns of both boys and girls when compared to controls, the timing and nature of the differences can vary between the sexes during development [23–25].

Behavior plays a critical role in the determination of obesity, especially physical activity[26], screen time[27, 28], eating behavior[29] and sleep habits[30]. Moreover, the clustering of more than one energy balance-related behavioral risk factor is known to be more deleterious to health than a single factor alone in both children[31–35] and adults[36, 37]. While those behaviors have been frequently investigated in the literature, to the best of our knowledge no study has investigated then together in the same sample in relationship to birth weight and sex.

The above evidence opens the possibility for the existence of a more complex expression of the low and high birth weight phenotypes. These individuals would not only have a Metabolic Thrifty Phenotype, expressed by their increased adiposity deposition with consequent higher cardiometabolic risk, as proposed by Barker et al[38, 39], but also what we are calling a Thrifty Eating or Thrifty Behavior Phenotype. This would be characterized by an increase in the intake of palatable foods and decrease energy expenditure in favor of a positive energy balance[13]. Obesity and cardiometabolic risk in this population would hence be a result not only of peripheral metabolic adaptations, but also from concomitant altered behaviors acting as mediators or adjuvants of these effects.

In the present study, we aimed to further explore this hypothesis by characterizing the associations between birth weight and different obesogenic behaviors across the sexes: screen time[40], physical activity[41], eating behavior[42] and sleep[30], elucidating possible points for more effective behavioral interventions aimed at both treatment and prevention.

## Methods

A sample of households from the Montreal Metropolitan area was selected from a large database of families who previously indicated their willingness to participate in academic research and were likely to have children in the targeted age group (6–12 years old). These families were contacted by telephone by an independent research firm and invited to take part in a survey about children’s eating and lifestyle habits on behalf of the researchers from McGill University. This survey was part one of a multi-component Brain-to-Society diagnostic study aiming to map children’s behaviors with regards to a multitude of factors, including their environment, eating habits, physical activity, and body mass indexes (BMI). Questionnaires were completed by children’s parents or guardians.

Respondents were first asked whether there were any children aged 6–12 years who lived in their households most of the time (>50% of the time in a regular week). If yes, the interviewer then proceeded to ask to speak with the parent or guardian who knew the child’s daily habits best. If more than one child between 6–12 years old was residing within the household, the parent/guardian was asked to answer all questions regarding the child who had the next birthday. Verbal consent was obtained from all participants who were mailed a $10 check for completing the 50-minute telephonic survey. Data collection was conducted between March and August 2013 and received ethical approval from McGill University’s Institutional Research Board(A09-M15-12A). All procedures performed in studies involving human participants were in accordance with the ethical standards of the institutional and/or national research committee and with the 1964 Helsinki declaration and its later amendments or comparable ethical standards.

### Measures

The survey included household demographic information, children’s anthropometrics (height and weight), and web-based questionnaires on sleeping, screen time (TV, computer) and physical activity time, and eating behavior. The eating behavior questionnaire was adapted from the Dutch Eating Behavior Questionnaire (DEBQ)[43], and was reported by the parents or caregiver. The questions were re-worded for parents to report on their child’s behavior and adapted to fit the cultural context and improve suitability for children. For instance, the term delicious was replaced for “yummy” and food sources like cafes replaced for corner stores (‘dépanneur’). The number of response options were also changed from five to three consistent with previous adaptations of the scale for children [44]. Internal consistency of this scale for the sample, assessed following the approach recommended by Gadermann and colleagues [45] for estimating reliability coefficients for ordinal data, was found to be within the acceptable to good range for external (Cronbach's Alpha = 0.74) and emotional (Cronbach's Alpha = 0.86) eating, although near acceptable for restrained eating (0.67)[42, 44, 46]. Sleep Time (hours per night) was obtained from the Children’s Sleep Habits Questionnaire[47]. Sports, Play, and Active Recreation for Kids (SPARK) Questionnaire developed by Sallis et al.[48] and questions from the School Health Action Planning and Evaluation Systems (SHAPES) Survey[49] were used to measure physical activity and screen time. Questions included a 7-day recall of how many hours and minutes the child had spent on vigorous and/or moderate physical activity and activities involving a screen. Examples of both vigorous and moderate physical activity and screen activities were detailed in the questionnaire and responses were asked for each day of the week.

The behavioral outcomes were Emotional, Restrained and External eating scores from the DEBQ questionnaire[46], physical activity (minutes per week), screen time (minutes per week) and hours of sleep (hours per night). The anthropometric outcome, BMI, was calculated following the formula weight(Kg)/height (cm)^2^, and normalized to age and sex (z-score) using WHO growth standards and methodology[50]. To normalize birth weight(g) to gestational age, sex for populational standards, we utilized the Birth Weight Ratio (BWR), that is the ratio between the infant birthweight and the sex-specific mean birthweight for each gestational age in weeks for the local population[51, 52].

### Statistical methods

The first set of analyses tested associations between BWR and behavioral outcomes across sexes. Physical activity, screen time, emotional eating and dietary restraint were all positively skewed and modelled using Poisson models; sleep time and external eating were modelled using linear regression. A second set of analyses tested the associations between BWR and zBMI before and after inclusion of the individual behaviors. For behaviors for which statistically significant associations were found with both BWR and zBMI, a formal mediation test using bootstrapping was conducted in MPLUS Version 8 (Los Angeles, CA: Muthén & Muthén). Birth Weight Ratio (BWR) was treated as a continuous variable and mean-centered prior to analyses. Baseline characteristic were analyzed using BWR divided in tertiles and One-Way ANOVA (Age) and Chi-square (Income and Ethnicity) was applied to the sample stratified by sex. To explore potential u-shaped relationships between BWR and outcomes linear and quadratic terms for BWR were included in all models. All models were adjusted for age, sex ethnicity and household income categorized into three categories (< 45,000; 45,000 to 65,000; >65,000 CAD). As stated in the introduction, we expect sex differences in obesity related behavioral factors, therefore sex-stratified analyses where conducted. Analyses were performed using SAS version 9.4 (SAS Institute Inc., Cary, NC, USA). In all analyses, statistical significance was set at 0.05.

## Results

Data were available for 460 children (230 boys and 230 girls). Characteristics of the sample are presented in Table 1 by birth weight tertiles.

**Table 1:** Characteristics of the sample.

	Boys (n = 230)							Girls (n = 230)						
BWR Tertile (range)	Lower (0.54 to 0.94)		Middle (0.95 to 1.08)		Upper (1.09 to 1.54)			Lower (0.65 to 0.94)		Middle (0.95 to 1.08)		Upper (1.09 to 1.76)		
	n = 78		n = 74		n = 78		p-value	n = 76		n = 80		n = 74		p-value
**Income (%)**														
>65K / year	52	(34.7%)	50	(33.3%)	48	(32.0%)	-	48	(32.4%)	49	(33.1%)	51	(34.5%)	-
45K to 65k / year	10	(28.6%)	10	(28.6%)	15	(33.3%)	-	15	(33.3%)	21	(46.7%)	9	(20.0%)	-
<45k / year	16	(35.6%)	14	(31.1%)	15	(33.3%)	0.815^a^	13	(35.1%)	10	(27.0%)	14	(37.8%)	0.255^a^
Age (SD)	9.1	(1.7)	9.3	(1.6)	9.3	(1.7)	0.540^b^	9.1	(1.7)	8.8	(1.7)	9.0	(1.7)	0.556^b^
**Ethnicity**														
Canadian	66	(84.6%)	64	(86.5%)	62	(79.5%)	-	58	(76.3%)	64	(80.0%)	63	(85.1%)	-
Other	12	(15.4%)	10	(13.5%)	16	(20.5%)	0.482^a^	18	(23.7%)	16	(20.0%)	11	(14.9%)	0.393^a^

SD: standard deviation.

^a^Pearson Chi-Square.

^b^One-Way ANOVA.

### Associations between birth weight and behavior

**Fig 1:**
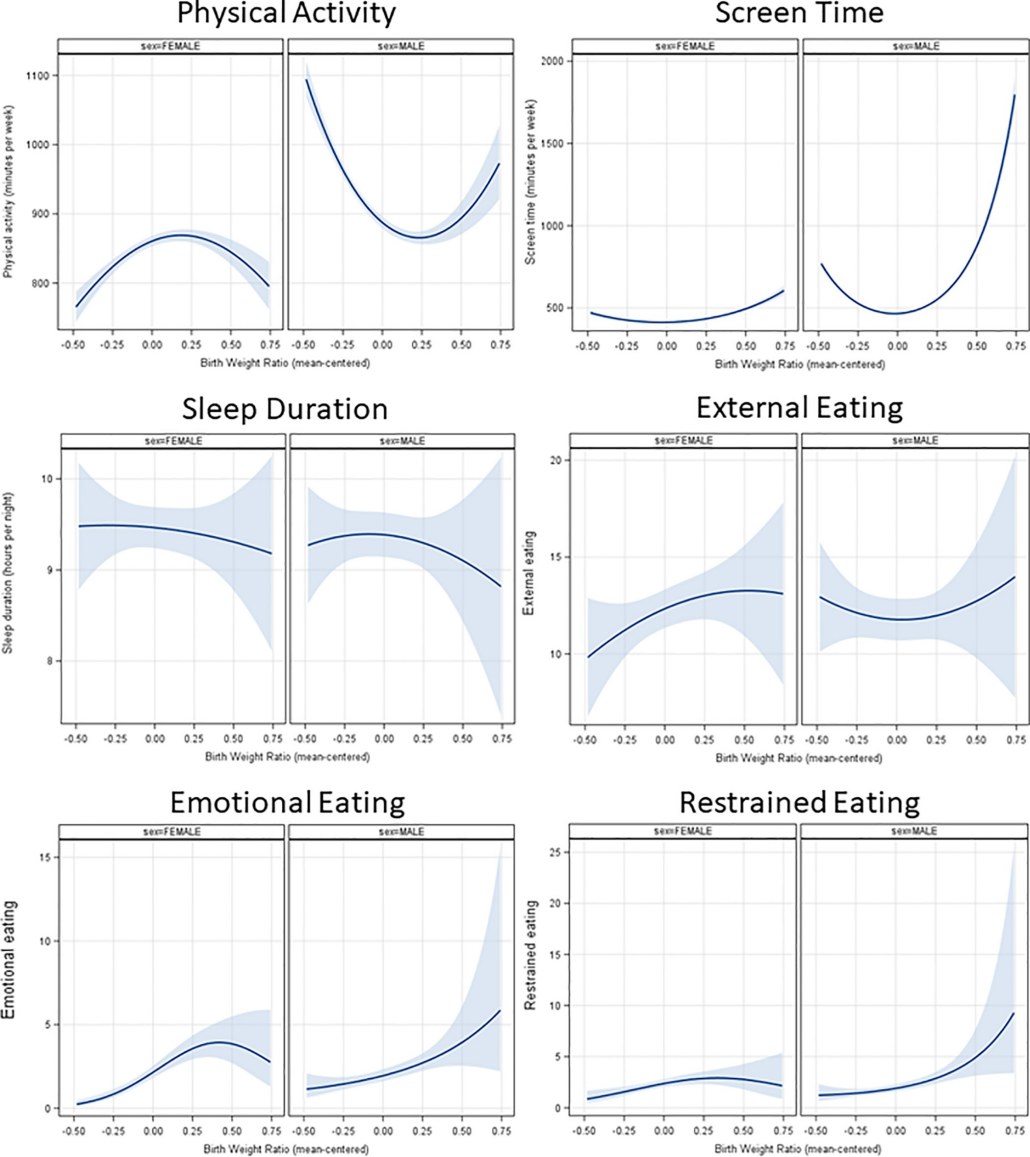
Fitted curves between birth weight ratio and health behaviors by sex. Figure showing fitted curves between Birth Weight Ratio and health behaviors by sex.

Results of analyses testing associations between BWR and health behaviors are reported in Table 2. Main effects results suggested the presence of curvilinear relationships between BWR and physical activity and screen time as indicated by significant quadratic terms. Emotional and restrained eating were found to be mostly linearly related to BWR. No statistically significant associations were found for sleep and external eating measures. Interactions between BWR terms and sex indicated significant interactions for physical activity, screen time, emotional and restrained eating. Fitted curves for boys and girls are shown in Fig 1. A U-shape relationship between BWR and physical activity was found for boys, in which lower BWR boys had the highest physical activity scores. This relationship was inverted for girls, with girls on either extreme of the BWR distribution having lower physical activity scores compared to girls with moderate BWR. A U-shape relationship was also found for screen time in boys, with screen time being highest in boys with higher birth weight ratios. This U-shape relationship was also found in girls, but with reduced magnitude. Sex-specific results for emotional and restrained eating suggested a mostly linear relationship between eating behaviors and BWR in boys. The same relationships were found to follow an inverted U-shape relationship in girls.

**Table 2:** Results of analyses testing associations between birth weight ratio (BWR) and behaviors stratified by sex.

Predictor		Physical Activity (95%CI)		Screen Time (95%CI)		Sleep Estimate(95%CI)	
		BOYS	GIRLS	BOYS	GIRLS	BOYS	GIRLS
**BWR**		-0.21 (-0.23,-0.18)***	0.01 (0.07,0.13)***	0.04 (0.01,0.018)**	<0.01 (-0.03,0.04)	-0.20 (-0.89,0.50)	-0.16 (-0.86,0.53)
BWR*BWR		0.50 (0.40,0.60)***	-0.26 (-0.35,-0.17)***	.96 (1.85,2.07)***	0.64 (0.54,0.74)***	-1.25 (-3.96,1.45)	-0.37 (-2.28,1.82)
**age**		-0.01 (-0.01,-0.01)***	-0.07 (-0.07,-0.07)***	0.10 (0.10,0.10)***	0.05 (0.05,0.05)***	-0.15 (-0.22,-0.08)***	-0.21 (-0.28,-0.15)
**Household income (> 65K as reference group)**	<45	-0.03 (-0.04,-0.02)***	0.05 (0.03,0.06)***	-0.04 (-0.05,-0.02)***	0.12 (0.11,0.14)***	-0.35 (-0.66,-0.04)*	-0.10 (-0.41,0.20)
	45-65K	-0.14 (-0.17,-0.13)***	-0.02 (-0.03,-0.01)**	0.22 (0.21,0.24)***	0.07 (0.05,0.08)***	-0.17 (-0.51,0.16)	-0.02 (-0.25,0.31)
**Ethnicity**		-0.05 (-0.06,-0.03)***	0.10 (0.09,0.12)***	0.08 (0.07,0.10)***	-0.39 (-0.41,-0.37)***	0.01 (-0.32,0.34)	-0.40 (-0.67,-0.12)**
		**Emotional eating (95%CI)**		**Restrained eating (95%CI)**		**External eating (95%CI)**	
		**BOYS**	**GIRLS**	**BOYS**	**GIRLS**	**BOYS**	**GIRLS**
**BWR**		1.23 (0.67,1.80)***	2.88 (2.16,3.60)***	1.45 (0.84,2.06)***	1.27 (0.60,1.95)**	-0.56 (-3.36,2.24)	3.30 (-0.05,6.65)
BWR*BWR		0.91 (-1.11,2.94)	-3.29 (-5.10,-1.48)**	1.35 (-0.81,3.51)	-1.90 (3.87,0.06)	3.60 (-7.28,14.48)	-2.65 (-12.37,7.07)
**age**		0.17 (0.11,1.23)***	0.08 (0.02,0.13)*	0.09 (0.03,0.16)**	0.19 (0.13,0.25)***	0.05 (-0.24,0.34)	-0.39 (-0.71,-0.08)*
**Household income (> 65K as reference group)**	<45	1.02 (0.79,1.23)***	0.01 (-0.24,0.27)	0.31 (0.06,0.56)*	0.10 (-0.17,0.37)	0.82 (-0.44,2.08)	0.62 (-0.82,2.07)
	45-65K	0.84 (0.60,1.08)***	0.30 (0.08,0.52)**	0.18 (-0.10,0.46)	0.35 (0.12,059)**	1.03 (-0.32,2.38)	-0.25 (-1.60,1.10)
**Ethnicity**		0.23 (0.00,0.45)*	0.42 (0.21,0.63)***	0.34 (0.10,0.59)**	0.54 (0.32,0.75)***	1.45 (0.13,2.77)*	1.04 (-0.28,2.36)

Physical activity, screen time, emotional eating and dietary restraint were modelled using Poisson models. Screen time and external eating were modelled using linear regression

*<0.05

**<0.01

***<0.0001

### Associations between birth weight ratio, obesogenic behaviors and BMI

**Fig 2:**
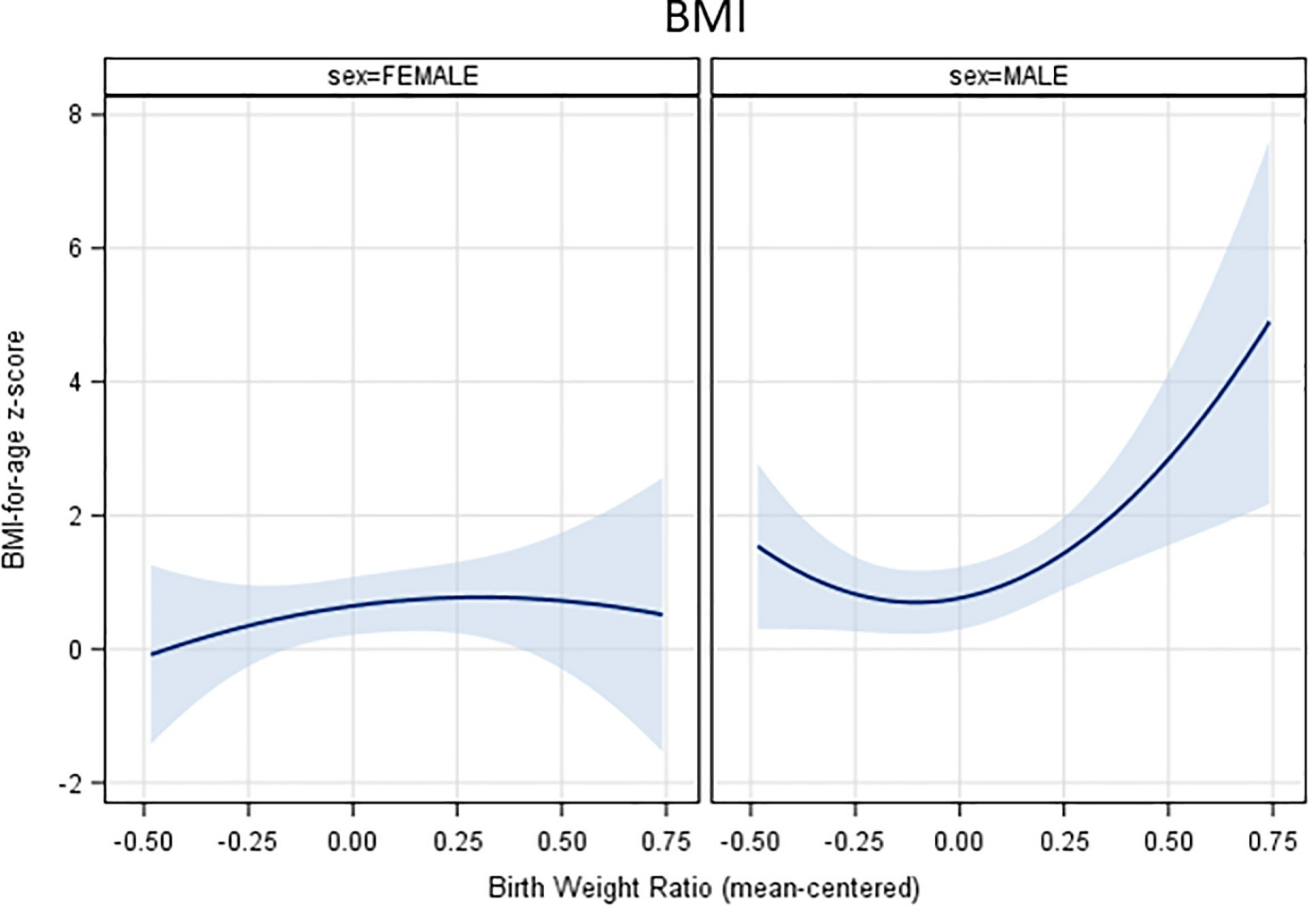
Fitted curves between birth weight ratio and zBMI in boys and girls.

Results of analyses testing the association between BWR and zBMI (reported in Table 2) suggest that the relationship was curvilinear and statistically significant with evidence of gender differences in the relationship. Sex-specific fitted curves and analyses (presented in Fig 2 and Table 3, respectively) indicated that the relationship was statistically significant and U-shaped in boys, with zBMI being highest in boys with high BWR, but relatively flat and non-significant for girls. Individual behaviors were added to the zBMI model (see Table 3) to explore potential mediation by behaviors. Only sleep (in boys and girls), and emotional and restrained eating (girls only) were found to be related to zBMI. Given that sleep was not related to BWR, only mediation by emotional and restrained eating in girls was tested. Results of indirect (mediated) effect testing using bootstrapping of the associations between both the linear and quadratic terms of BWR on zBMI through emotional and restrained eating suggested a possible mediation of the linear BWR term through emotional eating (Indirect effect: 0.345; 95%CI = 0.062, 0.629; P = 0.017). None of the other indirect effects reached statistical significance (P-values > 0.12).

**Table 3:** Results of models (estimates (95% CI)) testing associations between birth weight ratio and zBMI accounting for individual behaviors, by sex.

		No behavior		Physical Activity		Screen time		Sleep	
		BOYS	GIRLS	BOYS	GIRLS	BOYS	GIRLS	BOYS	GIRLS
**BWR**		1.25 (-0.06,2.56)	0.83 (-0.54,2.21)	1.22 (-0.08,2.54)	0.84 (-0.54,2.22)	1.24 (-0.06,2.56)	0.83 (-0.54,2.20)	1.18 (-0.11,2.48)	0.77 (-0.57,2.13)
BWR*BWR		6.07 (0.98,11.16)*	-1.23 (-5.22,2.76)	6.13 (1.04,11.21)*	-1.24 (-5.25,2.77)	6.07 (0.94,11.21)*	-1.41 (-5.39,2.57)	5.68 (0.65,10.70)*	-1.36 (-5.30,2.56)
**Behavior**		-	-	<0.01 (0.00,0.00)	0.00 (0.00,0.00)	<0.01 (0.00,0.00)	<0.01 (0.00,0.00)	-0.31 (-0.55,-0.07)*	-0.37 (-0.64,-0.15)**
**age**		0.02 (-0.12,0.15)	-0.02 (-0.15,0.11)	-0.02 (-0.12,0.15)	-0.02 (-0.15,0.11)	0.02 (-0.12,0.16)	-0.03 (-0.16,0.10)	-0.03 (-0.17,0.11)	-0.10 (-0.24,0.04)
**Household income (> 65K as reference group)**	<45	0.85 (0.16,1.34)*	0.09 (-0.50,0.69)	0.74 (0.15,1.33)	0.09 (-0.50,0.69)	0.75 (0.16,1.34)*	0.06 (-0.53,0.66)	0.64 (0.05,1.22)*	0.05 (-0.53,0.64)
	45-65K	0.49 (-0.14,1.12)	0.32 (-0.24,0.87)	0.47 (-0.16,1.11)	0.31 (-0.24,0.87)	0.49 (-0.15,1.12)	0.30 (-0.25,0.85)	0.43 (-0.19,1.06)	0.32 (-0.22,0.87)
**ethnicity**		0.40 (-0.22,1.01)	0.46 (-0.09,1.00)	0.39 (-0.22,1.00)	0.46 (-0.08,1.01)	0.39 (-0.22,1.01)	0.54 (-0.01,1.08)	0.40 (-0.21,1.00)	0.31 (-0.23,0.85)
**Predictor**				**Emotional eating**		**Restrained eating**		**External eating**	
				**BOYS**	**GIRLS**	**BOYS**	**GIRLS**	**BOYS**	**GIRLS**
**BWR**				1.16 (-0.16,2.47)	0.25 (-1.12,1.62)	1.02 (-0.30,2.34)	0.66 (-0.72,2.03)	1.24 (-0.07,2.55)	0.71 (-0.66,2.10)
BWR*BWR				5.96 (0.88,11.04)*	-0.82 (-4.71,3.06)	5.68 (1.62,10.75)*	-0.98 (-4.95,2.99)	6.10 (1.01,11.20)*	-1.13 (-5.19,2.85)
**Behavior**				0.03 (-0.03,0.10)	0.12 (0.06,0.18)***	0.08 (-0.01,0.17)	0.08 (-0.01,0.17)	-0.01 (-0.07,0.05)	0.03 (-0.02,0.09)
**age**				0.01 (-0.13,0.15)	-0.04 (-0.16,0.09)	0.01 (-0.13,0.14)	-0.05 (-0.18,0.08)	0.02 (-0.12,0.15)	-0.01 (-0.14,0.12)
**Household income (> 65K as reference group)**	<45			0.66 (0.06,1.27)*	0.09 (-0.49,0.67)	0.69 (0.11,1.28)*	0.08 (-0.52,0.67)	0.75 (0.16,1.35)*	0.10 (-0.50,0.70)
	45-65K			0.43 (-0.21,1.07)	0.24 (-0.31,0.78)	0.46 (-0.16,1.09)	0.26 (-0.30,0.81)	0.50 (-0.14,1.13)	0.32 (-0.23,0.88)
**ethnicity**				-0.37 (-0.24,0.99)	-0.35 (-0.18,0.88)	-0.34 (-0.28,0.95)	-0.37 (-0.18,0.91)	-0.41 (-0.21,1.03)	-0.42 (-0.13,0.96)

Physical activity, screen time, emotional eating and dietary restraint were modelled using Poisson models. Time and external eating were modelled using linear regression

*<0.05

**<0.01

***<0.0001

## Discussion

In this study we have found some distinct patterns of association between the continuum of birth weight and childhood obesogenic behaviors considering linear and quadratic distributions and sex[53]. Birth weight effects were more evident in boys, as compared to girls, especially for physical activity and screen time. These sex differences in behaviors and obesity risk are expected, and most certainly related to the many neurobiological dissimilarities between boys and girls—especially in terms of fat rebound timing, puberty, and differential growth patterns[21]—and gender associated effects, where physical activity is generally found to higher in boys[54, 55], especially when considering high intensity activities[56]. On the other hand, screen time has been less associated with sex differences[27, 28].

In analyzing the associations with BMI, we found that BMI had a u-shaped relationship with birth weight in boys. Several studies have indicated that high birth weight is associated with subsequently higher levels of body weight or obesity in childhood [57, 58], nevertheless, the evidence for a u-shaped (both extremes of birth weight) [59] or for the low birth weight side alone are more scarce[10]. One possible explanation could that low birth weight individuals have overall smaller bodies and the decreased lean mass would compensate the BMI increase[60]. Nevertheless, for the same BMI, the low birth weight individuals usually have more cardiometabolic complications[61, 62]. There is also some evidence suggesting an increased susceptibility to the beneficial effects of physical activity in the low birth weight individuals. Laaksonen et al. observed that a low size at birth was associated with hyperinsulinemia only in less active men[63], and likewise, Eriksson et al. found that they were strongly protected from glucose intolerance by regular exercise[64].

In boys both physical activity and screen time had a non-linear, U-Shaped distribution, meaning the extremes of the birth weight spectrum had higher reported physical activity and screen time. While increased physical activity is associated with better anthropometric and metabolic outcomes[27, 65], the increased screen time is associated with opposite effects[28]. This could represent a compensatory move, where increased sedentary time occurs in response to the excess energy expenditure related to physical activity, although some studies have shown that children can have high levels of both[26, 66], and that the mechanisms involved on the association between screen time and obesity may be not related to energy expenditure, but due to altered eating habits with increased intake of energy dense snacks and dinking more sugared drinks[67, 68]. Girls had the opposite direction for physical activity, but a similar pattern for screen time. As discussed above, the expression of sedentary/activity behavior may be sex-specific, a fact corroborated by Shakir et al, where sedentary behavior (video game playing) was positively related to obesity only in boys[69]. The effect on physical activity in girls, assumes an inverted U-shaped form, which is in the expected direction, especially taking into account the evidence that low birth weight individuals have reduced physical performance, including alterations in muscle mass and strength, muscle endurance and lower aerobic fitness later in life [60, 70–77]. Brutsaert *et al*, for instance, showed clear deficits in muscle strength and fatigue resistance in college-aged women with low ponderal index at birth[78]. Alterations in physical capacity may lead to reduced levels of physical activity[79, 80]. Screen time has been identified not only as a risk factor for obesity [1], but also for neurodevelopmental[2], psychological[3], behavioral and feeding alterations[4]. It has been reported also that neurocognitive functions are predictors of future patterns of weight gain in prospective studies [5], while compliance to healthier behaviors has been shown to be associated with a higher global cognition outcome[2]. The mutual influence of such factors makes the inference of causality very difficult, and even point-out to the possibility of mutual reciprocal effects, or perhaps still unidentified mediators

In terms of eating behavior, the association with birth weight assumed a more linear relationship, with emotional and restrained eating being positively related with birth weight. Emotional overeating refers to a tendency to overeat in response to negative emotions[81]. Findings on the relationship between emotional overeating and overweight are controversial. Some studies found no correlation[29, 82, 83], while others found a correlation with increased weight[84–87] as well as increased body fat percentage, waist and hip circumference[87]. Cortisol cord blood levels are similar in small and high birth weight individuals[88], but emotional eaters may have altered cortisol reactivity to stress, which induces overeating[89]. Restrained eating is defined as an attempted restriction of food intake[90]. Snoek *et al* showed a positive relationship between overweight and restrained eating in adolescents[29] and similar findings have been reported by others[82, 83]. Our results are in accordance to those described above and could explain the increased risk that children with higher birth weight have of becoming overweight in the future. One possible mechanism for such alteration might be related to alteration of the mesocorticolimbic dopaminergic pathways involved in the control of appetite[91, 92]. In fact, Eisenstein et al found that emotional eating was associated with striatal dopamine D2/D3 binding[93] and Volkow et al found that striatal dopamine response to foods was associated with restrained and emotional eating but not to external eating[94]. Also, in animal research, early life overnutrition has been found to increase dopaminergic precursors and to affect appetite for palatable foods[95, 96], similarly for prenatal undernutrition[11, 12, 97]. In general, low birth weight girls/women seem to be more vulnerable to the effects of prenatal programming of food preferences, showing increased preference for carbohydrates, impulsivity for sugar and emotional overeating when compared no normal birth weight counterparts[10, 13, 98]. However, other studies do not seem to find a sex effect[99], and in a subsample of the same cohort, we found a negative correlation between fat intake and BWR in boys, suggesting an increased preference for fat in the low birth weight side[19]. Therefore, while prenatal programming seems to modify eating patterns of both boys and girls when compared to controls, the timing and nature of the differences can vary between the sexes[24], fluctuating according to the adiposity rebound and body fat distribution during development [23].

Extensive literature from animal research has showed important effects of early life low and high birth weights on feeding behavior, appetite and physical activity[12, 95, 100–103]. A recent systematic review addressing the effects of birth weight on energetic metabolism associated behaviors in humans, such as physical activity, energetic intake, and some aspects of feeding behavior[104], did not find much evidence in the literature for such associations, except for decreased physical activity levels in both extremes of birth weight. However, this review did not explore the effects of birth weight on eating behaviors like feeding preferences, as measured by a differential intake of macronutrients, energy density or specific group of foods–such as fruits and vegetables—which is the most common finding in humans[10, 105, 106].

Sleep, eating behavior and cardiovascular control are closely related. It is known that birth weight, besides the influence on obesity and cardiovascular function, also influences the development of adequate sleeping patterns[9, 107]. Sleep is also associated to feeding behavior[108], adiposity[109], and metabolism[110, 111]. Such multidirectional effects have suggested a possible mediation pathway for sleeping into determining obesity, but in our case the number of sleeping hours was only associated with BMI, not birth weight.

Taking together, our results could suggest that, despite the similar risk for obesity and related diseases, Low and High birthweight individuals from different sexes might be cruising toward different pathways, making the identification of these differences critical for a more precise medical approach towards both prevention and treatment. Our study rises important findings about the relationship between birth weight and obesity, by including altered behavior in the equation. Moreover, the positive mediation found in this study places these altered feeding behavior as a candidate to be in the causal pathway towards obesity in this population. One possible mechanism for such alteration might be related to alteration of the mesocorticolimbic dopaminergic pathways involved in the control of appetite[91, 92].

Our study, nevertheless, has some limitations that have to be accounted for in the interpretations of the results. This is a cross-sectional analysis, therefore the relationships are associative, not causative. Our data analysis also treated the behaviors in isolation and future research should try to explore their interrelationship. The data collected are based in self-reported questionnaires, that although validated and carefully collected, always leave some potential for bias. Self-reported BMI is generally biased towards underreporting of obesity[112, 113], although in our case it would favor the null hypothesis. Moreover, especially in small sample sizes or underpowered studies, accuracy is more critical for categorization or clinical diagnosis, than it is for correlations. Our birth weight means, for instance, deviates only 1.6% for males and 3.3% for females from the average Canadian birth weights, normalized to gestational age and sex, while for BMI z-scores, are similar to contemporary research in Canada[114, 115]. The subscale restrained eating of the DEBQ questionnaire had an internal consistency of 0.67, which is slightly below the cutoff for acceptable, but emotional and external eating were in the acceptable range(0.86 and 0.74 respectively). Although our findings are not a definitive and conclusive response to the question, they rise important considerations about the different mechanisms involved in the pathway to obesity, highlighting the importance of behavioral, and individual brain eating responses to emotions and self-control in relationship to birthweight.

In summary, boys and girls born on the extremes of the normal range of birth weight are showing different phenotypical expression of behaviors while sharing the same risk of obesity. Therefore, the early life programming of vulnerability to obesogenic behaviors may take gender-specific behavioral causal pathways, opening perspectives of investigation, and prompting to the identification new strategic opportunities for prevention and treatment to yield better targeted prevention and treatment.

## Supporting information

S1 File Anonymized dataset. (ZIP)
